# Exploring associations between positive and negative valanced parental comments about adolescents’ bodies and eating and eating problems: a community study

**DOI:** 10.1186/s40337-022-00561-6

**Published:** 2022-03-24

**Authors:** Lucy M. Dahill, Natalie M. V. Morrison, Haider Mannan, Deborah Mitchison, Stephen Touyz, Kay Bussey, Nora Trompeter, Phillipa Hay

**Affiliations:** 1 Eating Disorder and Body Image Network, Western Sydney University, Campbelltown, Australia; 2 School of Medicine, Translational Health Research Institute, Western Sydney University, Locked Bag 1797, Campbelltown, Penrith, NSW 2715 Australia; 3 School of Medicine, Western Sydney University, Campbelltown, Australia; 4 Inside Out, University of Sydney, Sydney, Australia; 5 School of Psychological Science, Centre for Emotional Health, Macquarie University, Sydney, Australia; 6 Camden and Campbelltown Hospitals, Campbelltown, Australia

**Keywords:** Weight-related behaviours, Eating disorder, Body image concerns, Mental health, Psychological distress, Teenagers, Family, Teasing

## Abstract

**Background:**

Adolescence is a time of rapid emotional and physical development when foundational self-concepts (including beliefs about one’s weight and shape) are established. Parents are key influencers of adolescent beliefs and behaviours. This study aimed to investigate associations between perceived positive and negative parental comments on weight/shape and eating, with sons’ and daughters’ psychological distress and eating disorder cognitions (EDCs).

**Methods:**

A representative mixed-sex sample of 2204 Australian adolescents (12–19 years) from the EveryBODY Study completed an online survey exploring eating behaviours, psychological wellbeing and experiences of parental comments regarding weight, shape and eating behaviours.

**Results:**

Correlation analyses revealed that adolescents’ reports of perceived positive parental comments on shape/weight were significantly associated with lower psychological distress and EDCs only for daughters. All perceived negative parental comments on shape/weight or eating were associated with greater psychological distress and EDCs for both sons and daughters. In the final model of the regression analysis, only perceived parental negative shape/weight and maternal negative eating comments, adolescent stage and biological sex were significantly associated with EDCs. When known contributors such as BMI percentile and psychological distress were included in the regression model, adolescent stage and perceived negative paternal comments were no longer significantly associated with EDCs.

**Conclusions:**

Overall, results show perceived negative comments were associated with poorer adolescent mental health, both their specific EDCs and general distress. Findings highlight the importance of raising awareness of potential negative impacts within family systems of comments around weight/shape and eating in these key formative years.

*Trial Registration* The study was approved by the Macquarie University Human Research Ethics Committee (HREC 5201600312) and the New South Wales Department of Education.

**Supplementary Information:**

The online version contains supplementary material available at 10.1186/s40337-022-00561-6.

## Plain English summary

Adolescence is a time of rapid emotional and physical development when beliefs about one’s weight and shape are established. Parents are key influencers of adolescent beliefs and behaviours. This study aimed to consider associations between perceived positive and negative parental comments on weight/shape and eating, with sons’ and daughters’ psychological distress and behaviours associated with eating disorders. We found all perceived negative comments from either parent were associated with poorer adolescent mental health, both specific to behaviours associated with eating disorders and general distress. When we considered the strength of the relationship between parental comments and different influences such as biological sex, developmental stage, BMI percentile and psychological distress, we expected to find that BMI percentile and psychological distress would influenced the findings, and they did. When we considered the relationship with them included, perceived maternal negative comments continued to have a negative influence on behaviours related to eating disorders. Therefore, our findings highlight the importance of raising awareness of potential negative impacts within family systems of comments around weight/shape and eating in these key formative years.

**Supplementary Information:**

The online version contains supplementary material available at 10.1186/s40337-022-00561-6.

## Background

Adolescence is a time of dramatic changes in physical, social and emotional development [1, 2]. Foundations are set during these years for learned behaviours to engrain and ultimately project into lifelong habits [3–5], including the shaping of social-emotional regulation and eating behaviours and problems [6–8].

An extensive research base has found that the communication of messages via explicit or implicit means such as through media, peers, siblings and parents, influences Eating Disorder Cognitions (EDCs) such as fear of weight gain, body dissatisfaction, self-objectification [9–11]; and distress over weight / shape, as well as other features e.g., dysfunctional emotion regulation, low self-esteem, anxiety, avoidant coping strategies [12–14] and depression [14, 15]. All of these aspects individually, and certainly in combination, increase the likelihood for the development of eating disorders amongst adolescents [16]. In this research, we investigated specific EDCs of weight and/or shape overvaluation as these are defining aspects of self-view and self-regard. Weight/shape overvaluation is also a diagnostic criterion for anorexia nervosa and bulimia nervosa (DSM5) and common in other eating disorders [17].

Exploring the role played by parents in this circle of influence and their impact, is particularly important bearing in mind parents’ ongoing engagement with their child in terms of hours per week spent in their child's company, care-giving, the parent–child attachment dynamic being built, and the emotion regulation modelling provided by parents [2, 12]. It is understandable, therefore, to consider that parents contribute to their adolescents’ sense of security and connectedness [18], influence the emotional warmth and perceived safety of the home environment [19, 20], and contribute to their child’s psychological health over this timespan [21]. Conversely, criticisms and teasing of body weight and shape, or verbal encouragements of weight loss, can undermine the presumed quality of interaction of the parent–child dyad within the family system [7, 22, 23].

To date, the role of the parent–child dyad has proved complex to understand and is not generally well represented across aetiology models of prominence in the eating disorder field. Authors suggest parental influence may be more of a contributing factor rather than a sole cause and declines over the adolescent years as adolescence increases their sense of autonomy [19, 21]. However, while parental influence might be considered only as “contributory”, it has been posited that parental commentary that acts to confirm, unintentionally or with purpose, other negatively valanced messages (i.e., from the media or from peers in the Tripartite Influence Model) is powerful given parents are often considered to be the people who ‘know us best’ suggesting their words have broader influence [24–28].

The perceived valence, by adolescents, of their parents’ comments regarding weight, shape and eating is relevant because it could influence eating problems which are associated with eating disorders, the co-occurrence of which are prevalent during adolescence [29, 30]. A review of perceived negative parental comments, including teasing, with regards to weight/shape and/or eating during these formative years found that their presence may impact on the development of psychological distress (symptoms of anxiety/depression), as well as eating disorder symptoms [31]. Such weight, shape or eating comments appear to be common, however, their prevalence varies widely, from 12% [27] to 76% [32] according to participant sampling and the content of comments investigated.

We recently reported a representative study of the prevalence of valanced positive and negative comments regarding weight/shape/eating from mothers’ and father’s as perceived by their adolescent sons and daughters [33]. The study found that generally positive comments around weight and shape were reported by adolescents, with mothers more likely than fathers to be the source (78% and 51% respectively). However, adolescents also reported perceived negative comments regularly in regards to their weight and shape, with 37% of adolescents reporting these from their mothers and 28% reporting these from their fathers. Regularly receiving parental comments specific to eating behaviours were also reported (maternal = 70% and paternal = 44%) with the majority of these being viewed as positive as opposed to negative in nature. Clearly adolescents perceive their parents to make a considerable number of comments about their shape, weight and eating behaviours—however it was unclear from this prevalence study the impact of these comments on adolescents’ own eating beliefs, behaviours, and psychological wellbeing.

Early meta-analyses provided some insight about how the content of comments made about weight, shape and eating behaviours might have influenced wellbeing by reporting that “health-focused” conversations might be less detrimental than other less purposeful comments [34, 35] and in some cases such “healthy commentary” might even prove protective against EDCs [10, 36, 37]. However, this is contrasted by literature indicating that parental encouragement to lose weight [38] and negative comments [39] were associated with body dissatisfaction; including unhealthy strategies to lose weight [8, 40], extreme weight loss [39], dieting and eating and weight concerns [41]. Prospectively, weight-related teasing by parents was associated with increased risk for disordered eating [42].

More recently Gillison et al. [34] reported in their meta-analysis that both being encouraged to lose weight and being teased or criticised about weight by parents were associated with negative self-perceptions and more eating disorder cognitions (EDCs). This suggests that adolescents’ perception and the specific content of comments regarding weight, shape, and eating behaviours are important and warrants further investigation. What a parent perceives as their intention when they comment on their adolescents’ weight, shape and/or eating behaviours may be of little importance and instead it is how such comments are perceived by the adolescent that is important. For instance, Puhl and Himmelstein [43] reported that a mother might consider their weight or eating comment on their daughter’s weight to be positive (e.g., “you’ve certainly lost weight, you’re looking good”), but the daughter might hear it as a criticism (e.g., “you were overweight”) or an unnecessary comment that drives overvaluation in weight and shape [43]. Thus, research is needed to examine whether the valence of the comments received from parents makes a difference in terms of associations with indicators of disordered eating and wellbeing.

Gendered dyads within families influence the types of conversations about eating and weight/shape that take place. Such conversations have typically been gendered with boys being more attuned and or reactive to comments (such as muscle-ideation) from their fathers and girls attuned or reactive to mothers with comments (such as on thin-idealisation) due to gender role identification [36, 37, 44]. The content of such conversations is likely influenced by the parent’s own experiences of societal norms and the expectations around appearance that might have proven important during their own lives [45, 46]. The importance of considering parental weight, shape and eating behaviours commentary through a gendered lens is highlighted in view of findings that suggest female and male adolescents appear differentially impacted by their gendered parents’ comments. Whereas daughters’ disordered eating appears to be equally driven by comments from both parents, sons’ disordered eating predominantly arises when such comments are delivered by their father figure [47]. Social Cognitive Theory (SCT) provides a theoretical framework to account for the influence of modelling through environmental and personal factors, and their interaction on health behaviours [48, 49]. Literature investigating comment content (i.e., positively verses negatively valanced words) with a focus on the parent–child gender dyad is uncommon [39, 47]. However, as noted earlier, Dahill et al. [32] reported that daughters perceived significantly more negative weight/shape and eating comments from mothers, whereas sons perceived significantly more negative weight/shape comments from fathers, which again highlights the importance of further investigating associations of such gendered nuances.

Studies to date have also highlighted two important characteristics that have also limited the understanding of the impact of parental comments on weight, shape and eating behaviours amongst adolescents: (1) age inclusion limitations and (2) presence of confounding mental wellbeing variables. Firstly, at this time the literature within this field has demonstrated a tendency to focus on age via a specific period of adolescence such as early [24, 27, 39, 42, 50]; middle [32, 36, 37, 46, 51], or middle to late adolescence [35, 52]. Few studies have examined the whole of adolescence [39, 41], what stage comments are more likely to be reported and whether they are perceived as positive or negative. The most recent of these studies reported that the prevalence of parental comments varied with age. Specifically, negative comments were found to increase and positive comments to decreased with reported age. Whilst paternal positive comments on weight, shape and eating to daughters was protective, the older adolescents reported fewer perceived positive comments, and negative maternal comments on weight shape and eating with daughters reached significance [33]. This finding highlights the need to consider age as a key factor and acknowledgment that adolescence comprises a broad developmental age range within this field of study. Secondly, while several studies have controlled for potential confounding or moderating factors such as body mass index (BMI; kg/m^2^) or BMI percentile (BMI%ile), biological sex or age [27, 32, 36, 42, 51–55] very few have examined psychological distress as a covariate of eating disorder outcomes when looking at comment content (e.g., positively verses negatively valanced) and gender dyads (e.g., mothers and fathers with daughters and sons) [56]. Mental health problems and distress have previously been reported to be higher in girls than in boys [57].

### Objective

The main aim of the current study is to determine associations between perceived positive and negative weight/shape and eating comments from either gender parent, and daughter’s and son’s eating disorder cognitions (EDCs) and psychological health (K-10). Based on previous literature, theoretical frameworks and known prevalence of eating disorders cognitions in girls compared to boys, specific hypotheses tested were: (1) perceived negative comments from mothers will be associated with poorer psychological health and heightened EDCs among daughters; (2) negative paternal comments around weight and shape will be associated with poorer psychological health and heightened EDCs among sons; (3) the strength of a putative positive association between negative parental weight/shape or eating comments, and daughters psychological health and EDCs will increase with adolescent stage and (4) positive comments from either parent will have a positive association with their sons' and daughters' psychological health and EDCs. The research design also facilitated further exploration on other familial dyads (e.g., mother/son and father/daughter) and associations with psychosocial risk factors for EDCs. Whilst exploratory, we do include a broad hypothesis that the size of the effects of comments are expected to be greater in girls than boys [29].

## Method

### Participants

3239 Australian adolescents completed Wave 2 of the EveryBODY study [16], a longitudinal investigation of eating disorders and body image among Australian adolescents. This longitudinal project included examination of several potential risk factors, including in Wave 2, questions developed by the researchers pertaining to frequency of positive and negative comments about weight, shape and eating received by the adolescent from parents. Participants gave their informed consent to participate in the survey and were offered the opportunity to enter a prize draw for one of ten $100 vouchers following their participation. Data were collected via an online questionnaire completed during school time, under teacher supervision. Parental opt-out consent was used, whereby parents were provided information about the survey and could choose to withdraw their child from participating. In addition, all adolescents who participated provided assent to do so. The 8 participating schools which comprised of 4 government and 4 independent schools, were provided with a summary report on the wellbeing of their students based on the data provided by their students. Full recruitment processes have been described elsewhere, however, it is noteworthy that the participants were recruited from schools whose socio economic status was similar to the average Australian school [16]. The study was approved by the Macquarie University Human Research Ethics Committee (HREC 5201600312) and the New South Wales Department of Education. For the current study, all participants (aged between 12 and 19 years) who indicated they had a parent in their life, were an adolescent at the time of participation (defined by the World Health Organisation as between the age of 10 and 19 years) and had completed the questions related to parental comments, were included. In total, *n* = 952 participants were excluded for one of the following reasons: consent was not given, or implausible responses were recorded (*n* = 110); the individual did not indicate having at least one parent in their life (*n* = 105); they did not complete the parent comment questions (*n* = 737). Those who did not answer the questions on parental comments were more likely to be female, younger and of a higher BMI%ile. A further *n* = 83 participants were removed during data analyses due to the detection of outliers (discussed later in statistical methods). The final sample included 2204 (68% of the recruited pool) participants. See Additional file 1 for an accounting of this process and an evaluation of retained and excluded participant characteristics (Additional files 2, 3).

### Measures

#### Specific parental comment questions

Participants were asked, "Is your mother in your life?" Responses were binary (i.e., yes/no) yet, taking into account the non-binary make-up of our families, the following clarification was offered “if you have two mother figures in your life, please answer for the one you spend the most time with”. Participants who gave an affirmative response were asked the following 4 questions regarding maternal comments on (i) weight and shape and (ii) eating, with the following template: "How often does your mother comment positively (negatively) on your weight or shape (eating)?" Participants rated positive and negative comments regarding weight and shape, and regarding eating, on a 5-point scale with the following response options "never" (1), "rarely" (2), "sometimes" (3), "often" (4), and "all of the time" (5). These questions were then asked regarding comments from fathers or ‘father figures’ in their lives. There were 8 questions in total. See Additional file 4 for the full list of questions.

#### Eating disorder cognitions (EDCs)

Twelve items from the Weight Concern and Shape Concern subscales of the Eating Disorder Examination Questionnaire (EDEQ-WS) [58] were used to assess cognitive eating disorder symptoms/psychopathology, referred to in this study as eating disorder cognitions [59]. Participants are asked questions specifically related to weight and shape pathology over the past 4 weeks (28 days), such as “how dissatisfied have you been with your shape?”. The Likert scale was used to measure responses (0 = *Not at all* to 6 = *Markedly*). A mean of the 12 items was used to compute a global score, ranging from 0 to 6. The combined EDEQ-WS score has been used in several previous studies, including to define overall body image disturbance in the diagnosis of anorexia nervosa [16]. The EDEQ-WS has demonstrated good reliability amongst Australian adolescent boys and girls [60]. Internal consistency (Cronbach alpha) in other studies has been found to be 0.89–0.91 [61] and 0.90–0.94 [62]. The current study found the 12-item measure to have excellent consistency as indicated by a Cronbach alpha of 0.96.

#### Psychological distress

Frequency of anxiety and depressive symptoms during the past 4 weeks were assessed using the K-10 Psychological Distress Scale, which has demonstrated high clinical utility in predicting clinically significant levels of distress in diverse populations [63–65] and with adolescents [66]. Participants completed 10 items on how often they felt specific feelings (e.g., Tired out for no good reason”) in the last 28 days (4 weeks) on a 5-point Likert scale (1 = *None of the time* to 5 = *All of the time*). Scores range from 10 to 50, with higher scores indicating higher levels of distress. The current study found the K-10 measure to have excellent internal consistency as indicated by a Cronbach alpha of 0.93. Reliability in other studies has been found to be 0.94 [67].

#### Body Mass Index (BMI) percentile

Participants were asked to self-report their height and weight measurements which were then used to calculate BMI (kg/m^2^). BMI was then converted to sex-adjusted BMI percentiles (BMI%iles). Percentiles were calculated in line with Centers for Disease Control and Prevention Guidelines (2017).

### Demographic information

From participants, the following demographics were collected: Age (years and months), biological sex (male, female), school year (7 to 12), self-report weight and height, own ethnicity and mother and father ethnicity.

### Statistical methods

SPSS version 27 was used in all analyses. We explored the quantitative data using Shapiro test for normality which revealed that the data were not normally distributed; therefore, we calculated medians and associated IQRs. The quantitative variables included eating disordered cognitions (i.e., EDEQ-WS) and psychological distress (i.e., K10). For checking internal consistencies of parental comments questions, we used Cronbach’s alpha. A minimum of 0.70 for Cronbach’s alpha is considered to be internally consistent. Bootstrapping analyses are considered a robust empirical solution to violations of normality [68, 69]. As the quantitative variables were non-normal, bootstrapping analyses were conducted to provide corrected *p*-values and confidence intervals for the linear regressions. To detect outliers and influential cases, multivariate assumptions were first addressed by inspecting the z score residuals to identify any unusual relations between the individual independent variables and the dependent variable. Secondly, Mahalanobis’ distance scores were then evaluated to determine whether any usual response patterns existed across the independent variables. Any individual participant data highlighted as exceeding 3.29 in the first instance or having a Mahalanobis’ distance critical value greater than 32.91 in the second instance were removed from further analysis in accordance with the recommendations of Tabachnick and Fidell [70]. Following outlier removal this process was repeated, with the data of any additional identified participants also removed, until no further outliers were identified according to these processes. Details of this process are outlined in Additional file 1. Alpha was adjusted to *p* < 0.01 to eliminate familywise error. As the proportion of missing data in any analyses did not exceed 12% no imputations were made [71].

Bivariate correlations were used to determine associations between parental comments, adolescent stage, biological sex, BMI%ile, EDEQ-WS and K10 using Pearson’s Correlation and the number of bootstrap samples set to 1000 for constructing 95% confidence intervals using the bootstrap percentile method [69]. Cohen’s [72] rationale for interpreting effect sizes was used to guide interpretation (Small 0.10, Medium 0.30, Large 0.50, Very Large 0.70) [72]

Hierarchical regression was used to examine associations with parental weight/shape and eating comments, adolescent stage and biological sex as regressor variables and EDEQ-WS scores. A second regression was then conducted to ascertain whether the unique contributions revealed for the regressor variables of particular interest in this research, were similarly valuable and impactful when considered alongside the well documented influence of psychological distress (i.e., K10 scores) and weight (i.e., BMI%ile) on eating disorders.

In this study, age was used as a continuous variable for the descriptive analysis of sons and daughters, and then considered as a categorical variable in the regression by stratified adolescent stages: that is, school year (grades) 7 and 8 (mapping to early adolescence (40.9%)), school year (grades) 9–10 (mapping to mid-adolescence (42.3%)) and school year (grades) 11 and 12 (mapping to late adolescence (16.8%)). This grouping was based on the rationale that peer cohorts mature together (e.g., socially and academically) and thus two students of the same age will be less comparable if they are in two different year groups than two students within the same year group. This grouping has proved appropriate in previous research [2, 29, 33]. We used the partial F test to assess the significance of the interaction between BMI%ile and adolescent stages.

## Results

### Descriptive data

The 2204 participants represented students from four independent and four government secondary schools in New South Wales, Australia. Participants had a mean age of 14.84 years (*SD* = 1.50). We considered sons and daughters based on biological sex due to students from one school only being allowed a question “what is your gender” with response options “male or “female” (*n* = 450). The number of students who nominated “non-gender specific” was 10; therefore, it was decided to retain the larger cohort based on identified biological sex as "male" or "female" for this study. In the final sample, 53% of the sample identified as female and 47% identified as male. Within the results section onwards, we use the terms “sex” and “sons” and “daughters” as opposed to “male” and “female” to reflect the decision to use the “biological sex” (sex assigned at birth) data and the dyadic nature of the child-parent relations captured in this study. Median Body Mass Index (BMI) percentile was 52.80 (*IQR* = 24.83–77.25). Adolescent participants and their parents' racial/ethnic backgrounds were as follows: Adolescents—83.1% Australian, 2.3% European, 12.0% Asian, 1.1% Oceania/Pacific Island, 1.4% other. Fathers—65.4% Australian, 5.7% European, 21.3% Asian, 2.9% Oceania/Pacific Island, 3.4% other. Mother—65.5% Australian, 4.9% European, 22.5% Asian, 3.3% Oceania/Pacific Island, 3.5% other. Because of the small number of youth reporting North or South American, African and 'other' parentages, these participants are grouped and coded together as 'other'. Participant demographic details are further summarised in Table 1. Sons were older compared to daughters [*t*(2285) = 2.093, *p* = 0.04, *d* = 1.50] and sons had a higher average BMI%ile compared to daughters [*t*(2233) = 3.34, *p* < 0.001, *d* = 30.26]. As expected, daughters had higher average scores for eating disorder cognitions [EDEQ-WS; *t*(2280) = − 19.397, *p* < 0.001, *d* = 1.59] and psychological distress [K10; *t*(2285) = − 12.419, *p* < 0.001, *d* = 9.38].

**Table 1:** Summary of participant demographic information (N = 2204)

	Sons		Daughters		Total population	
	N	Median (IQR)	N	Median (IQR)	N	Median (IQR)
Age	1042	14.92 (13.58–16.08)	1162	14.67 (13.50–15.83)	2204	14.75 (13.58–15.92)
BMI%ile	1004	57.35 (26.05–82.58)	1148	50.10 (24.55–73.00)	2152	52.80 (24.83–77.25)
Eating Disorder Symptomology (EDEQ-WS)	1038	0.29 (0.00–1.08)	1161	1.58 (.50–3.58)	2199	0.75 (.08–2.42)
Psychological Distress (K10)	1042	15.00 (12.00–21.00)	1162	20.00 (14.00–29.00)	2204	17.00 (13.00–25.00)

*BMI%ile* Body Mass Index percentile (kg/m^2^), *EDEQ-WS* Eating Disorder Examination Questionnaire-Weight/Shape Sub Scale, *K10* Kessler 10-item scale, *N/n* participant number (all/sub-group), *IQR* inter quartile range

### Correlation analyses

#### Positive comments from mothers and fathers to daughters and sons on weight, shape and eating

Table 2 displays the correlations between positive comments made by mothers and fathers on their daughters’ and sons’ weight, shape and eating, and the outcomes of interest, disordered eating cognitions (as captured by the EDEQ-WS) and psychological distress (as captured by the K10).

**Table 2:** Positive weight/shape and eating parent comment associations to daughters and sons on Eating Disorder Symptoms (EDEQ-WS) and psychological distress (K10) measures

EDEQ-WS	Maternal		Paternal	
	Correlation coefficient	Significance	Correlation coefficient	Significance
	All comments *N* = 1949		All comments *N* = 1949	
Positive W/S	.010	.653	− .049	.029
Positive eating	− .030	.184	− .038	.092
	Daughters comments *n* = 1037		Daughters comments *n* = 1037	
Positive W/S	− .140	< .001	− .104	< .001
Positive eating	− .098	.002	− .110	.001
	Sons comments *n* = 912		Sons comments *n* = 912	
Positive W/S	.040	.223	.016	.640
Positive eating	.016	.626	.045	.172

K10	Maternal		Paternal	
	Correlation coefficient	Significance	Correlation coefficient	Significance
	All comments N = 1949		All comments N = 1949	
Positive W/S	− .065	.004	− .121	< .001
Positive eating	− .107	< .001	− 102	< .001
	Daughters comments *n* = 1037		Daughters comments *n* = 1037	
Positive W/S	− .188	< .001	− .177	< .001
Positive eating	− .157	< .001	− .159	< .001
	Sons comments *n* = 912		Sons comments *n* = 912	
Positive W/S	− .031	.344	− .062	.060
Positive eating	− .086	.010	− .049	.142

Pearson Correlation and bootstrap *p* value

Unless otherwise noted bootstrap results are based on 1000 bootstrap samples

*W/S* Weight Shape comments, *EDEQ-WS* Eating Disorder Examination Questionnaire-Weight/Shape Sub Scale, *SOBBS* Self-Objectification Beliefs and Behaviours Scale, *FNE* Fear of Negative Evaluation Scale, *K10* Psychological Distress

As can be seen, positive weight/shape comments from either parent were negatively associated with daughters’ K10 and EDEQ-WS scores, and reached significance with small effect sizes. This suggests that the presence of positive comments by either parent in this data, was associated with lower psychological distress and EDCs for daughters. No positive comments reached significance for sons.

#### Negative comments from mothers and fathers to daughters and to sons on weight, shape and eating

As seen in Table 3, negative parental comments were also correlated with daughters’ and sons’ scores on the EDEQ-WS and K10.

**Table 3:** Negative weight/shape and eating parent comment associations to daughters and sons on Eating Disorder Symptoms (EDEQ-WS) and psychological distress (K10) measures

EDEQ-WS	Maternal		Paternal	
	Correlation coefficient	Significance	Correlation coefficient	Significance
	All comments *N* = 1949		All comments *N* = 1949	
Negative W/S	.379	< .001	.232	< .001
Negative eat	.315	< .001	.200	< .001
	Daughters comments *n* = 1037		Daughters comments *n* = 1037	
Negative W/S	.415	< .001	.296	< .001
Negative eat	.379	< .001	.240	< .001
	Sons comments *n* = 912		Sons comments *n* = 912	
Negative W/S	.291	< .001	.268	< .001
Negative eat	.199	< .001	.184	< .001

K10	Maternal		Paternal	
	Correlation coefficient	Significance	Correlation coefficient	Significance
	All comments N = 1949		All comments N = 1949	
Negative W/S	.311	< .001	.199	< .001
Negative eat	.277	< .001	.178	< .001
	Daughters comments *n* = 1037		Daughters comments *n* = 1037	
Negative W/S	.340	< .001	.255	< .001
Negative eat	.317	< .001	.203	< .001
	Sons comments *n* = 912		Sons comments *n* = 912	
Negative W/S	.222	< .001	.184	< .001
Negative eat	.194	< .001	.157	< .001

Pearson Correlation and bootstrap *p*-value

Unless otherwise noted bootstrap results are based on 1000 bootstrap samples

*W/S* Weight Shape comments, *EDEQ-WS* Eating Disorder Examination Questionnaire-Weight/Shape Sub Scale, *SOBBS* Self-Objectification Beliefs and Behaviours Scale, *FNE* Fear of Negative Evaluation Scale, *K10* Psychological Distress

Results show that all negative weight/shape and eating comments from either parent towards their daughter or son were significantly associated with EDEQ-WS and K10 scores, with small to medium effect sizes. This suggests that the presence of negative comments was associated in this data with heightened presence of EDCs and psychological distress.

### Regression analysis

The previously conducted correlational analyses provided a correlation matrix which was inspected for all explanatory variables and their relationship with the primary outcome of eating disorder cognitions (see Table 4).

**Table 4:** Pearson Correlations and bootstrap *p* values between Eating Disorder Symptoms, Biological Sex, Adolescent Stage, Maternal and Paternal negative weight shape, and Maternal and Paternal negative eating comments, median level and IQR (N = 1949)

	Variables	*Median*	*IQR*	1	2	3	4	5	6	7	8	9	10	11
1	EDEQ -WS	.75	.08–2.42	–	.391**	.139**	.009	.377**	− .053	.232**	− .031	.311**	− .038	.197**
2	Biological Sex	2.00	1.00–2.00	.391**	–	− .021	.197**	.100**	.014	− .057	.052	.089**	.029	.008
3	Adol. Stage	3.00	1.00–3.00	.139**	− .021	–	− .066	.149**	− .086**	.069**	− .093**	.102**	− .142**	.035
4	Mat Pos WS	3.00	2.00–4.00	.009	.197**	− .066	–	− .068**	.540	− .010	.446**	.017	.374**	.037
5	Mat Neg WS	1.00	1.00–2.00	.377**	.100**	.149**	− .068**	–	− .013	.495**	− .007	.482**	.010	.349**
6	Pat Pos WS	2.00	1.00–3.00	− .053	.014	− .086**	.540**	− .013	–	.071**	.381**	− .017	.551**	.075**
7	Pat Neg WS	1.00	1.00–2.00	.232**	− .057	.069	− .010**	.495	.071**	–	.045	.388**	.118**	.625**
8	Mat Pos Eat	3.00	1.00–3.00	− .031	.052	− .093**	.446**	− .007**	.381**	.045	–	.074**	.616**	.100**
9	Mat Neg Eat	2.00	1.00–3.00	.311**	.089**	.102**	.017	.482**	− .017	.338**	.074**	–	.056	.525**
10	Pat Pos Eat	2.00	1.00–3.00	− .038	.029	− .142**	.374**	.010**	.551**	.118**	.616**	.056	–	.196
11	Pat Neg Eat	1.00	1.00–3.00	.197**	.008	.035	.037	.349**	.075**	.625**	.100**	.525**	.196**	–

*EDEQ-WS* Eating Disorder Examination Questionnaire-Weight/Shape Sub Scale, *Adol.* adolescent stage, *N* number included, *SD* standard deviation, *IQR* inter quartile range. *Mat Pos WS* maternal positive weight/shape comments, *Mat Neg WS* maternal negative weight/shape comments, *Pat Pos WS* paternal positive weight/shape comments, *Pat Neg WS* paternal negative weight/shape comments, *Mat Pos Eat* maternal positive eating comments, *Mat Neg Eat* maternal negative eating comments, *Pat Pos Eat* paternal positive eating comments, *Pat Neg Eat* paternal negative eating comments

**p < .01. Unless otherwise noted bootstrap results are based on 1000 bootstrap samples

The findings suggested that all explanatory variables shared significant relationships with the outcome variable (i.e., EDEQ-WS scores) with the exception of maternal positive weight shape and eating comments. Two categories of maternal positive commentary were not correlated with the EDEQ-WS scores in the ‘ALL adolescents’ correlations, but they were of importance in the gendered split for daughters compared to sons depicted previously. As such, we included all explanatory variables, including maternal positive comments, in the regression modelling.

In the first instance, we explored the relationship between adolescent stage and age and did not find a statistical significance between the two when entered into the model. We further hypothesized the effect BMI%ile is moderated by the stage categories. The average effect of BMI%ile when interacting with stage changed from *ß* = 0.002 to. *ß* = 006 as adolescents reported comments at mid-stage or late-stage, with early-stage adolescent being the reference. We found the effect of BMI%ile on the dependent EDEQ-WS variable increases however, the effect of BMI%ile is not moderated by adolescent stages as the increase is not significant (*p* = 0.16).

Therefore, regression analyses were conducted to ascertain the nuanced relationships between the explanatory variables of adolescent biological sex (i.e., daughters and sons), adolescent schooling stage (i.e., grades 7 and 8, grades 9 and 10, and grades 11 and 12), and the positive or negative maternal and paternal comments with respect to weight/shape and eating and the criterion variable of eating disorder cognitions (as measured by the EDEQ-WS) all of which were the focus of the current study. The outcomes of the regression model are presented in Table 5.

**Table 5:** Results of Bootstrap Linear Regression of perceived maternal and paternal negative weight shape and eating comments, biological sex and adolescent stage on the dependent variable eating disorder cognitions (EDEQ-WS) (N = 1994)

Variables	B	SE	Bias	*BCa 95% CI* B		
				LL	UL	*p*
Biological Sex	1.209	.065	− 9.56	1.088	1.341	< .001
Adolescent Stage	.097	.021	.000	.058	.137	< .001
Mat Pos W/S	− .014	.031	− 6.19	− .072	.048	.642
Mat Neg W/S	.374	.048	.000	.280	.467	< .001
Mat Pos EAT	− .035	.034	− .001	− .094	.030	.308
Mat Neg EAT	.201	.037	.000	.128	.270	< .001
Pat Pos W/S	− .033	.035	− .002	− .104	.030	.351
Pat Neg W/S	.210	.054	.000	.108	.314	< .001
Pat Pos EAT	− .030	.037	.000	− .104	.047	.394
Pat Neg EAT	− .026	.044	.000	− .118	.058	.546

*Mat* Maternal, *Pat* Paternal, *W/S* Weight Shape Comments, *EAT* Eating comments, *B* Unstandardized coefficients, *SE* Standard Error, *BCa* Bootstrap for Coefficients correcting for skew based on 100 bootstrap samples, *LL* lower level, *UL* upper level, *p* significance

Results of the multiple linear regression indicated that there was a collective significant association between biological sex, adolescent stage, maternal and paternal negative weight shape comments and maternal negative eating comments. Results indicated that the model itself was significant [*R* = 0.552, *R*^*2*^ = 0.305, adjusted *R*^2^ = 0.301, *F*(10) = 87.02, *p* < 0.001] and accounted for 30% of the variance on EDEQ-WS. The individual explanatory variables were examined further revealing that the majority of included variables were each making unique contributions to the model. The most influential factors for associations included, in descending order, biological sex (*ß* = 0.370, *p* =  < 0.001), Maternal negative weight/shape comments (*ß* = 0.207, *p* =  < 0.001), Maternal negative eating comments (*ß* = 0.144, *p* =  < 0.001), Paternal negative weight/shape comments (ß = 0.112, *p* =  < 0.001), Adolescent stage (ß = 0.086, *p* =  < 0.001). Paternal positive weight/shape, and eating comments, and paternal negative eating comments did not make unique contributions to the model.

The second regression analysis was conducted to ascertain whether these findings were robust following the inclusion of well-documented influencing factors of mental wellbeing (i.e., indicated by K10 scores) and weight (i.e., indicated by BMI%ile). The outcomes of the regression model are presented in Table 6.

**Table 6:** Results of Bootstrap Regression of perceived maternal and paternal negative weight shape and eating comments, biological sex, adolescent stage, BMI percentile and K10 on the dependant variable eating disorder cognitions (EDEQ-WS) (N = 1994)

Variables	B	SE	Bias	*BCa 95% CI* B		
				LL	UL	*p*
BMI%ile	.11	.001	9.78	.009	.012	< .001
K10	.86	.004	6.35	.079	.094	< .001
Biological sex	.843	.057	.000	.728	.956	< .001
Adolescent stage	.032	.018	− .001	− .001	.062	.069
Mat Pos W/S	− .007	.026	− .001	− .061	.043	.765
Mat Neg W/S	.217	.040	.000	.137	.295	< .001
Mat Pos EAT	.025	.030	.000	− .031	.085	.391
Mat Neg EAT	.096	.031	.000	.039	.154	< .001
Pat Pos W/S	.010	.029	.001	− .051	.071	.735
Pat Neg W/S	.102	.045	.002	.009	.197	.023
Pat Pos EAT	− .005	.031	.002	− .065	.048	.903
Pat Neg EAT	− .029	.037	.000	− .100	.044	.432

*BMI%ile* Body Mass Index (kg/m^2^) percentile, *K10* Kessler 10-item scale, *Mat* Maternal, *Pat* Paternal, *W/S* Weight Shape Comments, *EAT* Eating comments, *B* Unstandardized coefficients, *SE* Standard Error, *BCa* Bootstrap for Coefficients based on 100 bootstrap samples, *LL* lower level, *UL* upper level, *p* significance

Results of the multiple linear regression indicated that the inclusion of the additional variables (i.e., K10 and BMI%ile) did enhance the overall predictive power of the model [*R* = 0.735, *R*^*2*^ = 0.540, adjusted *R*^*2*^ = 0.538, *F*(12) = 189.70, *p* < 0.001]. However, the inclusion of these variables also altered the explanatory value of several variables that were identified in the previous model. Specifically, similar to the previous analysis, paternal positive weight shape comments and paternal positive eating comments did not contribute any unique variance to the model; however, in this second model we can also see that both adolescent stage and paternal negative weight shape comments were no longer significant contributors. On the other hand, the remaining variables of biological sex, maternal negative weight shape and maternal negative eating comments in combination with the newly added BMI%ile and K10 all have unique contributions. Overall, this adjusted model accounted for 54% of the variance of EDEQ-WS scores. The individual explanatory variables were examined further and indicated K10 is the biggest contributor to movement in the model, K10 (*ß* = 0.491, *p* =  < 0.001), followed by biological sex (*ß* = 0.257, *p* =  < 0.001), BMI%ile (*ß* = 0.195, *p* =  < 0.001), Maternal negative weight/shape comments (*ß* = 0.120, *p* =  < 0.001), Maternal negative eating comments (*ß* = 0.069, *p* =  < 0.001).

## Discussion

The current study investigated sons’ and daughters’ perceived positive and negative weight/shape and eating comments from mothers and fathers, and their associations with eating disorder cognitions, adjusting for psychological distress. All perceived negative parental comments when considered independently, were significantly associated with eating disorder cognitions (EDCs) and psychological distress in sons and daughters. Perceived negative maternal comments, particularly on their child’s weight and shape, were the most important with regards to presence of EDCs when controlling for biological sex and adolescent stage with perceived paternal negative comments on weight shape also having an association. Further, these results mostly held when controlling for factors known to be strongly associated with EDCs, i.e. BMI%ile and psychological distress. However, perceived paternal comments about eating and adolescent stage were no longer associated with EDC’s for sons nor daughters in the presence of BMI%ile and psychological distress.

Perceived negative parental comments and daughters and sons.

In this study, all perceived negative comments, except those from fathers about eating, were found to be associated with poorer daughter’s psychological health and heightened EDCs and mothers perceived negative weight/shape comments contributed almost as much as biological sex. Regression analysis found fathers perceived negative eating comments were no longer independently associated with EDC’s when BMI%ile and psychological distress were considered. When we consider the highly complex environment adolescents live within, the influence of modelling, the family system and dyads they are a part of, these associations between parents and their sons and daughters become worthy of greater understanding. The greater association of perceived maternal negative comments to daughters, is supported by social cognitive theory and prevalence research finding daughters perceive more negative comments than sons about their weight, and the source of these comments is more likely to be from their mother [33, 39, 44, 73, 74]. Further, the association with daughters’ poorer psychological health and heightened EDCs adds to the literature that mothers have the potential to influence their daughters’ wellbeing and view of their weight and shape [23–25, 27, 28, 34, 36, 37].

Perceived negative paternal and maternal comments around weight and shape were found to have a detrimental association with sons’ psychological health and EDCs. Regression analysis further supported the hypothesis. This is in line with previous research considering the influence of parental comments on sons and associations with psychological health and EDCs [36, 42, 44, 47, 52, 53, 75, 76]. Specifically, disordered eating in sons was found to be more prevalent when comments were provided by their father figures [47]. In the present study, sons had a higher BMI%ile than daughters, and this could have influenced how sensitive sons were to perceived negative comments as well as challenged their father’s perception of weight, shape and eating behaviours of their sons [44]. Further mixed-method and longitudinal research with both adolescents and their parents would be needed to understand these complex relationships.

Research has found adolescence to be a time when foundational self-concepts, including beliefs about weight and shape are established [6–8]. Findings in this study of a positive association between perceived negative parental weight/shape or eating comments, and daughters’ psychological health and EDCs increasing with adolescent stage are consistent with the finding that frequency was found to increase with adolescent stage [33]. However, when BMI%ile and psychological distress were considered alongside adolescent stage the latter lost its’ significance. The findings do suggest that further research should explore if adolescents place less value on negative comments as they get older and whether, as Bussey and Bandura posit, ‘differential modes of influence operate in concert or counteractively’ [44].

Parental research draws together the necessity to consider perception of negative comments from three perspectives. Firstly, parents may not mean their comments to be heard in the way they are received. When there is a duality of positive and negative, we consider words are either intended as positive or negative and perceived as positive or negative. Yet, this fails to account for the influence of non-verbal communication such as body language, intent, tone and context. For example, what may have been intended as a positive comment (e.g., “you’ve certainly lost weight, you’re looking good”), can be heard by a daughter who is anticipating a negative comment as unnecessary or criticism [43]. This could have been intended as a healthy conversation, but the perceived non-verbal cues could be interpreted by the adolescent as confirming a bias, a history, or pre-conception they have about the intention behind the comment. Further, it has been found that if a parent’s comment is influenced by their own experience of weight shape or eating ideals then such conversations may come with an unconscious bias, hence influencing the intent behind the comment which could be felt by the adolescent [45, 46]. Parental comments, the environment, personal factors and modelling of behaviours and ideas have the potential to influence heterosocial models of interaction [7, 27, 44, 46, 47]. Rodgers and Chabrol’s (2009) review of literature proposes parents’ attitudes towards body shape and eating do have a modelling effect on attitudes amongst adolescents and illustrates the importance of considering mothers and fathers as important sources of influence in their sons and daughters lives [7]. Direct comments can be more confronting and have been found to be perceived as teasing, criticism rather than encouragement to control or lose weight [7].

The second perspective for consideration is the foundations upon which the parent’s words are falling. If the son or daughter has low self-worth, or is vulnerable to influence from external sources outside the parent-adolescent dyad, such as peers or media, then a parent’s, particularly a mother’s, seemingly innocent and positively intended comment could result in confirmation bias and parents being blamed inadvertently for being the source of an offending comment [23, 36, 37]. Paternal comments to sons and daughters are under-researched compared to mother dyads, however, our findings suggest that although their influence held less value and therefore maternal comments have an overriding power, paternal comments were also a significant influence on EDCs [74] These gendered dyads should be explored further to consider the different modes of influence.

The finding in this study that paternal comments were no longer associated with sons or daughters eating disorder cognitions once BMI%ile and psychological distress were introduced to the model, lends itself to illustrate the third perspective. This is that negative comments coming from both parents appeared to have a cumulative negative impact on emotional health that is greater than that from a single parent, as illustrated by Eisenberg et al. [53]. The challenge presented for research in the field of family environment and eating disorders in part stems from the position not to blame parents, but rather see them as part of the different modes of influence and therefore part of the solution. This study does not seek to blame parents, rather, to posit that without considering the intention behind parents’ words, we will not understand why words have the influence they do on adolescent perception. Where the current study looked at maternal and paternal comments separately or as dyads, future studies might start to look at the interplay of two parents and how to support the triad relationship of parental commentary in family systems and EDCs. Research is also needed that considers the parents’ perception as well as the child’s perception, comments from siblings and qualitative studies which explore the adolescent’s experience of their parent’s comments.

### Positive parental comments

In this exploratory aspect to the research, overall, no perceived positive parental comments were associated with the child’s EDCs or psychological status. However, perceived positive comments made by parents on their child’s weight and shape, and perceived positive comments from mothers on their child’s eating, were found to be associated, albeit weakly, with lower levels of EDCs and psychological distress in daughters. Positive comments could be interpreted as more “health-focused” or they may be in the context of a more supportive situation, which have been found to be less detrimental [34, 35] and support other research as a potential protective influence for EDCs. The perceived positive valence for paternal comments in the early correlations, may support research that eating disorder symptoms in daughters may elicit a protective factor from fathers [75, 77] or be reflective of a more positive maternal relationship which could be protective [44]. Further, there are communication styles and dyads within families that contribute to adolescents’ sense of security and connectedness [18, 44]. Of note, this supports previous research that suggests healthy eating conversations have a protective nature and are associated with more ‘healthy’ weight control behaviours and lower levels of EDCs [36, 78]. The present study does not support Kluck’s finding that mothers’ encouragement was a stronger predictor of ED problems than father’s encouragement [79]. However, in this study, the intended perceived positive comment did not appear to act as a protective factor for EDCs when perceived negative comments were also present. This suggests it may be wiser for parents to ‘say nothing’ than to offer what is intended as a balance of positive and negative comments.

The strengths of this study are the large and representative sample of both boys and girls from 4 independent and 4 government schools, and their perception of comments from both parents. Further, we assessed both weight/shape as well as eating comments and used validated instruments for eating disorder cognitions and distress. Limitations included the cross-sectional design (thus directions of association could not be examined), and that weight and height were self-reported and that the recency of perceived comments was not specified. We acknowledge using biological sex is a limitation, future studies should use gender and determine responses based on the period of life for those who have changed gender in formative years. The later stage adolescents with higher BMI%ile were less likely to provide data and yet these were the participants hypothesized to be most vulnerable to comments. This could have resulted in under-reporting by these adolescents and affected the effect size of this finding. Also, whilst significant, the differences and effect sizes were small to medium therefore warrants further exploratory analysis as other factors such as the presence of social support from the parent maybe moderating the associations. Finally, whilst there was an attempt to capture the non-binary view of families in the parent comment questions by clarifying that it could be a “mother” or “father” figure, it would have been preferable to capture if the adolescents were also reporting on a step-parent.

## Conclusion

This study found that both mothers’ and fathers’ perceived comments on their child’s weight/shape and eating were associated with their adolescent child’s mental health in regard to EDCs and psychological distress. Associations were complex, greatest for perceived negative comments and from mothers, and mixed with regards to perceived positive comments. Further research is needed to understand these associations and guide families in ways to communicate issues around body image and eating using qualitative methods to explore further why words are perceived negatively by one person and potentially the same words may be perceived neutrally or positively by another. Longitudinal research is indicated to consider the direction (and potential bidirectionality) of associations and causal inferences. However, the findings suggest that public health and clinical interventions targeting improved communication around weight/shape and eating in family systems in these key foundational years may reduce eating disorder symptoms and the risk of eating disorders.

## Supplementary Information

**Additional file 1.** Multivariate Assumption Checks. A detailed outline of the multivariate process check

**Additional file 2: Table 7**. Pearson Correlations and bootstrap *p*-values between Eating Disorder Symptoms, Biological Sex, BMI percentile, K10, Adolescent Stage, Maternal and Paternal negative weight shape, and Maternal and Paternal negative eating comments, median level and IQR and included cohort (N = 1949). This is the Pearson correlation analysis with BMI percentile and K10 included for only included cohort

**Additional file 3: Table 8** Pearson Correlations and bootstrap *p*-values between Biological Sex, Eating Disorder Symptoms, Adolescent Stage, Maternal and Paternal positive and negative weight shape, and Maternal and Paternal negative eating comments, mean level and standard deviation. (N = 2077). This is the Pearson Correlations no BMI percentile and K10, with the full study participants for comparison purposes

**Additional file 4.** Specific Parent Comment Questions. The original questions exploring adolescent perception of positive and negative parental comments on weight shape and eating

## Data Availability

Deidentified data are available for collaborative projects from the author D.M. upon request, subject to approval from the authors’ institutional ethics committee.

## Abbreviations

EDCs: Eating Disorder Cognitions
BMI: Body Mass Index
BMI percentiles: BMI%iles
EDEQ-WS: Eating Disorder Examination Questionnaire-weight concern and shape concern subscales
K-10: Psychological Distress Scale
IQR: Interquartile range
