# Supplementary material for: Exploring associations between positive and negative valanced parental comments about adolescents’ bodies and eating and eating problems: a community study
Source: J Eat Disord. 2022 Mar 24;10:43. doi: 10.1186/s40337-022-00561-6 (PMC8953043; doi:10.1186/s40337-022-00561-6)
Supplement: Supplementary file 1 — Additional file 1. Multivariate Assumption Checks. A detailed outline of the multivariate process check [file 40337_2022_561_MOESM1_ESM.docx]

Additional File 1

Multivariate Assumption Checks

EDEQ-WS scores were used as the dependent variable by which associations and patterns between all the independent variables of interest were evaluated, including; Biological Sex, Adolescent Stage, BMI percentile (BMI%ile), K10 scores, Maternal positive comments on weight/shape, Maternal positive comments on eating, Maternal negative comments on weight/shape, Maternal negative comments on eating, Paternal positive comments on weight/shape, Paternal positive comments on eating, Paternal negative comments on weight/shape, Paternal negative comments on eating.

Multivariate assumptions were first addressed by inspecting the z score residuals to identify any unusual relations between the individual independent variables and the dependent variable. Secondly, Mahalanobis’ distance scores were then evaluated to determine whether any usual response patterns existed across the independent variables. Any individual participant data highlighted as exceeding 3.29 in the first instance or having a Mahalanobis’ distance critical value greater than 32.90949041 in the second instance were removed from further analysis in accordance with the recommendations of Tabachnick and Fidell (1). Following outlier removal this process was repeated, with the data of any additional identified participants also removed, until no further outliers were identified according to these processes.

To commence the data of 2,287 participants was screened for multivariate assumption violations. In the first round of multivariate assumption checking 12 cases exhibiting unusual relationships between the IVs and the DV (IV-DV) were identified along with a further 30 showing unusual relationships between the IVs (IV-IV). In the second round a further 23 were removed (IV-DV=7, IV-IV=16), in the third round 8 newly identified cases were removed (IV-DV=3, IV-IV=5), in the fourth round 6 further cases were removed (IV-DV=1, IV-IV=5), in the fifth round 4 further cases were removed (IV-DV=0, IV-IV=4). In the sixth round of multivariate assumption checking no cases were identified using either identification method suggesting all irregularities and assumptions violations were resolved through the removal of 83 cases in total.

To provide reassurance that the removal of the multivariate outliers was the most appropriate treatment correlational analyses were conducted firstly with the proposed excluded cases included, and secondly with the proposed excluded cases removed. The outcomes of these analyses, and the latter reported within the body of this study in table 4 suggested the proposed outliers played an influential role in establishing the relationships between the variables of interest. For this reason, the removal of the 83 cases was deemed an appropriate treatment for these outliers. This resulted in the data for 2204 being retained for further analysis.

The participant data of 83 participants represents only 0.04% of the original data set which is therefore unlikely to introduce bias into a sample of this size (2). The data removed was screened for any systematic underlying characteristics revealing that across the main demographic items of interest, being biological sex (𝜒2(1,N=2287) =.027, p=.870, Cramer’s V=.870), adolescent stage (𝜒2(2, N=2287)=2.176, p=.337, Cramer’s V=.337) and BMI%ile (t(2,233)=.349, p=.727).

**References**

1. Tabachnick BG. Using multivariate statistics. 5th ed. Fidell LS, editor. Boston: Boston : Pearson/Allyn & Bacon; 2007.

2. Lodder P. To Impute or not Impute: That’s the Question. 2014.
