# Supplementary material for: Exploring associations between positive and negative valanced parental comments about adolescents’ bodies and eating and eating problems: a community study
Source: J Eat Disord. 2022 Mar 24;10:43. doi: 10.1186/s40337-022-00561-6 (PMC8953043; doi:10.1186/s40337-022-00561-6)
Supplement: Supplementary file 2 — Additional file 2: Table 7. Pearson Correlations and bootstrap p-values between Eating Disorder Symptoms, Biological Sex, BMI percentile, K10, Adolescent Stage, Maternal and Paternal negative weight shape, and Maternal and Paternal negative eating comments, median level and IQR and included cohort (N = 1949). This is the Pearson correlation analysis with BMI percentile and K10 included for only included cohort [file 40337_2022_561_MOESM2_ESM.docx]

**Additional File 2 – Table 7**

*Pearson Correlations and bootstrap p-values between Eating Disorder Symptoms, Sex, BMI percentile, K10, Adolescent Stage, Maternal and Paternal negative weight shape, and Maternal and Paternal negative eating comments, median level and IQR and included cohort (N=1949)*

|  | Variables | *Median* | *IQR* | 1 | 2 | 3 | 4 | 5 | 6 | 7 | 8 | 9 | 10 | 11 | 12 | 13 |
| --- | --- | --- | --- | --- | --- | --- | --- | --- | --- | --- | --- | --- | --- | --- | --- | --- |
| 1 | Sex | 2.00 | 1.00-2.00 | - | -.054 | .266 | .391^**^ | -.024 | .198^**^ | .100^**^ | .015 | -.058 | .054 | .083^**^ | .029 | .008 |
| 2 | BMI | 52.80 | 24.83-77.25 | -.054 | - | .075 | .246^**^ | .108^**^ | -.011 | .123^**^ | .002 | .120^**^ | -.047 | .097^**^ | -.045 | .089^**^ |
| 3 | K10 | 17.00 | 13.00-25.00 | .266^**^ | .075^**^ | - | .639^**^ | .133^**^ |  | .311^**^ | -.121 | .199^**^ | -.107^**^ | .277^**^ | -.102^**^ | .178^**^ |
| 4 | EDEQ -WS | .75 | .08-2.42 | .391^**^ | .246^**^ | .639^**^ | - | .135^**^ | .010 | .379^**^ | -.049 | .232^**^ | -.030 | .315^**^ | -.038 | .200^**^ |
| 5 | Adol. Stage | 3.00 | 1.00-3.00 | -.024 | .108^**^ | .133 | .135^**^ | - | -.061 | .154^**^ | -.088^**^ | .071^**^ | -.093^**^ | .103^**^ | -.143^**^ | .036 |
| 6 | Mat Pos WS | 3.00 | 2.00-4.00 | .198^**^ | -.011 | -.065 | .010 | -.061 | - | -.080^**^ | .541^**^ | -.016 | .449^**^ | .008 | .375^**^ | .033 |
| 7 | Mat Neg WS | 1.00 | 1.00-2.00 | .100^**^ | .123^**^ | .311 ^**^ | .379^**^ | .154^**^ | -.080^**^ | - | -.017 | .487^**^ | -.009 | .488^**^ | .008 | .341^**^ |
| 8 | Pat Pos WS | 2.00 | 1.00-3.00 | .015 | -.002 | -.121^**^ | -.049 | -.088^**^ | .541^**^ | -.017 | - | .071^**^ | .379^**^ | -.025 | .555 | .068^**^ |
| 9 | Pat Neg WS | 1.00 | 1.00-2.00 | -.058 | .120^**^ | .199^**^ | .232^**^ | .071^**^ | -.016 | .487^**^ | .071^**^ | - | .040 | .341^**^ | .119 | .621^**^ |
| 10 | Mat Pos Eat | 3.00 | 1.00-3.00 | .029 | -.047 | -.102^**^ | -.030 | -.093^**^ | .449^**^ | -.009 | .379^**^ | .040 | - | .065 | 615^**^ | .094^**^ |
| 11 | Mat Neg Eat | 2.00 | 1.00-3.00 | .083^**^ | .097^**^ | .277^**^ | .315^**^ | .103^**^ | .008 | .488^**^ | -.025 | .341^**^ | .065 | - | .053 | .528^**^ |
| 12 | Pat Pos Eat | 2.00 | 1.00-3.00 | .029 | -.045 | -.102^**^ | -.038 | -.143^**^ | .375^**^ | .008 | .555^**^ | .119^**^ | .615^**^ | .053 | - | .198^**^ |
| 13 | Pat Neg Eat | 1.00 | 1.00-3.00 | .008 | .089^**^ | .178^**^ | .200^**^ | .036 | .033 | .341^**^ | .068^**^ | .621^**^ | .094^**^ | .528^**^ | .198^**^ | - |
| *Note*. BMI – Body Mass Index; EDEQ-WS - Eating Disorder Examination Questionnaire – Weight/Shape Sub Scale; K10 – Psychological Distress; Adol. = adolescent stage; N = number included; SD = standard deviation; M = mean; Mat Pos WS = maternal positive weight/shape comments; Mat Neg WS = maternal negative weight/shape comments; Pat Pos WS = paternal positive weight/shape comments; Pat Neg WS = paternal negative weight/shape comments; Mat Pos Eat = maternal positive eating comments; Mat Neg Eat = maternal negative eating comments; Pat Pos Eat = paternal positive eating comments; Pat Neg Eat = paternal negative eating comments  ***p* < .01. Unless otherwise noted bootstrap results are based on 1000 bootstrap samples | | | | | | | | | | | | | | | | |
