# Supplementary material for: Exploring associations between positive and negative valanced parental comments about adolescents’ bodies and eating and eating problems: a community study
Source: J Eat Disord. 2022 Mar 24;10:43. doi: 10.1186/s40337-022-00561-6 (PMC8953043; doi:10.1186/s40337-022-00561-6)
Supplement: Supplementary file 3 — Additional file 3: Table 8 Pearson Correlations and bootstrap p-values between Biological Sex, Eating Disorder Symptoms, Adolescent Stage, Maternal and Paternal positive and negative weight shape, and Maternal and Paternal negative eating comments, mean level and standard deviation. (N = 2077). This is the Pearson Correlations no BMI percentile and K10, with the full study participants for comparison purposes [file 40337_2022_561_MOESM3_ESM.docx]

**Additional File 3 - TABLE 8.**

*Bootstrap Pearson Correlations between Sex, BMI, Psychological Distress, Eating Disorder Symptoms, Adolescent Stage, Maternal and Paternal positive and negative weight shape, and Maternal and Paternal negative eating comments, mean level and standard deviation. (N=2077)*

|  | Variables | *Median* | *IQR* | 1 | 2 | 3 | 4 | 5 | 6 | 7 | 8 | 9 | 10 | 11 |
| --- | --- | --- | --- | --- | --- | --- | --- | --- | --- | --- | --- | --- | --- | --- |
| 1 | EDEQ -WS | .75 | .08-2.50 | - | .384^**^ | .127^**^ | .003 | .342^**^ | -.062^**^ | .209^**^ | -.038 | .281^**^ | -.030 | .189^**^ |
| 2 | Sex | 2.00 | 1.00-2.00 | .384^**^ | - | -.012 | .187^**^ | .077^**^ | .003 | -.071^**^ | .044 | .077^**^ | .019 | .000 |
| 3 | Adol. Stage | 3.00 | 1.00-3.00 | .127^**^ | -.012 | - | -.064^**^ | .135^**^ | -.092^**^ | .034 | -.088^**^ | .087^**^ | -.148^**^ | .010 |
| 4 | Mat Pos WS | 3.00 | 2.00-4.00 | .003 | .187^**^ | -.064^**^ | - | -.052 | .532^**^ | .013 | .440^**^ | .024 | .347^**^ | .036 |
| 5 | Mat Neg WS | 1.00 | 1.00-2.00 | .342^**^ | .077^**^ | .135^**^ | -.052 | - | .004 | .460^**^ | .018 | .499^**^ | .045^**^ | .337^**^ |
| 6 | Pat Pos WS | 2.00 | 1.00-3.00 | -.062^**^ | .003 | -.092^**^ | .532^**^ | .004 | - | .098^**^ | .371^**^ | .011 | .534^**^ | .095^**^ |
| 7 | Pat Neg WS | 1.00 | 1.00-2.00 | .209^**^ | -.071^**^ | .034 | .013 | .460^**^ | .098^**^ | - | .051 | .338^**^ | .141^**^ | .609^**^ |
| 8 | Mat Pos Eat | 3.00 | 1.00-3.00 | -.038 | .044 | -.088^**^ | .440^**^ | .018 | .371^**^ | .051 | - | .087^**^ | .584^**^ | .111^**^ |
| 9 | Mat Neg Eat | 2.00 | 1.00-3.00 | .281^**^ | .077^**^ | .087^**^ | .024 | .499^**^ | .011 | .338^**^ | .087^**^ | - | .092^**^ | .511^**^ |
| 10 | Pat Pos Eat | 2.00 | 1.00-3.00 | -.030 | .019 | -.148^**^ | .347^**^ | .045^**^ | .534^**^ | .141^**^ | .584^**^ | .092^**^ | - | .228^**^ |
| 11 | Pat Neg Eat | 1.00 | 1.00-3.00 | .189^**^ | .000 | .010 | .036 | .337^**^ | .095^**^ | .609^**^ | .111^**^ | .511^**^ | .228^**^ | - |
|  | *Note*. BMI – EDEQ-WS - Eating Disorder Examination Questionnaire – Weight/Shape Sub Scale; Adol. = adolescent stage; N = number included; SD = standard deviation; M = mean; IQR = inter quartile range. Mat Pos WS = maternal positive weight/shape comments; Mat Neg WS = maternal negative weight/shape comments; Pat Pos WS = paternal positive weight/shape comments; Pat Neg WS = paternal negative weight/shape comments; Mat Pos Eat = maternal positive eating comments; Mat Neg Eat = maternal negative eating comments; Pat Pos Eat = paternal positive eating comments; Pat Neg Eat = paternal negative eating comments  ***p* < .01. Unless otherwise noted bootstrap results are based on 1000 bootstrap samples | | | | | | | | | | | | | |
