# Supplementary material for: Exploring associations between positive and negative valanced parental comments about adolescents’ bodies and eating and eating problems: a community study
Source: J Eat Disord. 2022 Mar 24;10:43. doi: 10.1186/s40337-022-00561-6 (PMC8953043; doi:10.1186/s40337-022-00561-6)
Supplement: Supplementary file 4 — Additional file 4. Specific Parent Comment Questions. The original questions exploring adolescent perception of positive and negative parental comments on weight shape and eating [file 40337_2022_561_MOESM4_ESM.docx]

**Additional File 4 - Specific Parent Comment Questions as part of The EveryBODY Study**

Is your mother in your life?          yes/no

If yes, participants were asked to rate the following questions on a 5-point scale with the following response options "never" (1), "rarely" (2), "sometimes" (3), "often" (4), and "all of the time" (5).

How frequently did your mother positively comment on your body weight/shape (e.g., “you’ve got lovely legs”)?

How frequently did your mother negatively comment on your body weight/shape (e.g., “you really need to lose weight”)?

How frequently did your mother comment positively on your eating (e.g., “Great to see you eating salad!”)?

How frequently did your mother comment negatively on your eating (e.g., “You shouldn’t eat so much of that”)?

Is your father in your life?          yes/no

If yes participants were asked to rate the following questions on a 5-point scale with the following response options "never" (1), "rarely" (2), "sometimes" (3), "often" (4), and "all of the time" (5).

How frequently did your father positively comment on your body weight/shape (e.g., “you’ve got lovely legs”)?

How frequently did your father negatively comment on your body weight/shape (e.g., “you really need to lose weight”)?

How frequently did your father comment positively on your eating (e.g., “Great to see you eating salad!”)?

How frequently did your father comment negatively on your eating (e.g., “You shouldn’t eat so much of that”)?

How supported did you feel by others in your life when you received negative comments around your body weight/shape (e.g. did they support you or speak up for you)?

How frequently did you feel supported by others in your life when you received negative comments around your body weight/shape (e.g. did they support you or speak up for you)?
